# Cost-sensitive learning strategies for high-dimensional and imbalanced data: a comparative study

**DOI:** 10.7717/peerj-cs.832

**Published:** 2021-12-24

**Authors:** Barbara Pes, Giuseppina Lai

**Affiliations:** Dipartimento di Matematica e Informatica, Università degli Studi di Cagliari, Cagliari, Italy

**Keywords:** Cost-sensitive learning, Class imbalance, High-dimensional data analysis, Feature selection, Random forest

## Abstract

High dimensionality and class imbalance have been largely recognized as important issues in machine learning. A vast amount of literature has indeed investigated suitable approaches to address the multiple challenges that arise when dealing with high-dimensional feature spaces (where each problem instance is described by a large number of features). As well, several learning strategies have been devised to cope with the adverse effects of imbalanced class distributions, which may severely impact on the generalization ability of the induced models. Nevertheless, although both the issues have been largely studied for several years, they have mostly been addressed separately, and their combined effects are yet to be fully understood. Indeed, little research has been so far conducted to investigate which approaches might be best suited to deal with datasets that are, at the same time, high-dimensional and class-imbalanced. To make a contribution in this direction, our work presents a comparative study among different learning strategies that leverage both feature selection, to cope with high dimensionality, as well as cost-sensitive learning methods, to cope with class imbalance. Specifically, different ways of incorporating misclassification costs into the learning process have been explored. Also different feature selection heuristics have been considered, both univariate and multivariate, to comparatively evaluate their effectiveness on imbalanced data. The experiments have been conducted on three challenging benchmarks from the genomic domain, gaining interesting insight into the beneficial impact of combining feature selection and cost-sensitive learning, especially in the presence of highly skewed data distributions.

## Introduction

In the last decades, an increasing number of real-world applications have produced datasets with a huge dimensionality, *i.e.,* with a very large number of features. Biomedical data analysis, text mining and sensor-based data analysis are just some examples of application fields where the data instances can be represented in a very large feature space. Besides posing severe requirements in terms of computational resources, the high dimensionality may have a negative impact on the predictive performance of machine learning algorithms (the so-called “curse of dimensionality” issue) (Bolón-Canedo, Sánchez-Maroño & Alonso-Betanzos, 2015) and may also hinder the interpretability and the applicability of the induced models. The use of proper techniques to reduce the data dimensionality is then of utmost importance, as recognized by a vast amount of scientific literature in the field (Saeys, Inza & Larranaga, 2007; Khalid, Khalil & Nasreen, 2014; Dessì & Pes, 2015a; Tadist, Najah & Nikolov, 2019; Hambali, Oladele & Adewole, 2020). In particular, feature selection (Guyon & Elisseeff, 2003) has proven to be very effective in such a context, enabling to obtain faster, more accurate and more understandable predictors.

On the other hand, the high dimensionality often comes in conjunction with other issues embedded in the nature of data. In the context of supervised learning tasks, one of such issues is the imbalance in the class distribution (Branco, Torgo & Ribeiro, 2016; Fernández et al., 2018a), which may strongly degrade the generalization ability of traditional classification algorithms. Indeed, they are typically designed to minimize the overall prediction error, without distinguishing between different types of errors, and this may result in poor performance on the minority class(es). Despite being more difficult to recognize, however, rare instances can carry precious knowledge on the domain of interest and are often the most interesting/important from an application viewpoint (He & Garcia, 2009; Krawczyk, 2016).

Both the issues mentioned above, namely the high dimensionality and the class imbalance, have been extensively studied in the data mining and machine learning communities but, in most cases, they have been considered independently, as separate problems, without investigating their combined effects. Indeed, a limited amount of research has focused on learning strategies specifically conceived to cope with both issues simultaneously, *e.g.*, (Blagus & Lusa, 2010; Maldonado, Weber & Famili, 2014; Shanab & Khoshgoftaar, 2018; Zhang et al., 2019; Pes, 2020), and there is a need for more studies that systematically investigate the extent to which the methods so far proposed for handling class imbalance and reducing the data dimensionality can be effectively combined.

In this regard, a number of papers have recently explored the integration of feature selection and sampling-based data-balancing methods (Blagus & Lusa, 2013; Khoshgoftaar et al., 2014; Yin & Gai, 2015; Gao, Khoshgoftaar & Napolitano, 2015; Huang et al., 2021), suggesting that such a hybrid approach may be useful in some scenarios and also discussing and evaluating different integration strategies (*e.g.*, whether feature selection should be used before or after data sampling). On the other hand, less attention has been given to the integration of feature selection and cost-sensitive learning which is a potentially useful strategy that deserves more investigations (Feng et al., 2020; Pes, 2021).

To give a contribution in this field, our paper presents a comparative study among different learning strategies that properly combine feature selection, to deal with high dimensionality, and cost-sensitive learning methods, to deal with class imbalance. Essentially, cost-sensitive learning involves assigning different misclassification costs to the different classes, based on their importance for the task at hand, and then building a model capable of minimizing the total cost rather than the total number of errors, as in traditional classification. Although cost-sensitivity can be introduced directly into the learning algorithm, by modifying its design in an ad hoc way, there is also an interest in meta-learning approaches that can convert any existing method into a cost-sensitive one (Fernández et al., 2018a). This can be achieved by acting on the weights of the instances at the training stage or properly setting the probability threshold to classify new instances (Ling & Sheng, 2010). In this work, we aim to investigate the extension of such a meta-learning framework, so far applied in the context of low dimensional classification tasks (López et al., 2013), to hybrid learning strategies that also incorporate a feature selection step. Specifically, we study the impact of introducing cost-sensitivity at the feature selection stage or at the model induction stage under different cost settings and in conjunction with different selection methods. Such methods have been chosen as representatives of different paradigms and heuristics (*filter* and *embedded* methods, *univariate* and *multivariate* approaches), in order to assess and compare their effectiveness on imbalanced data.

As a representative case study, we focus on the analysis of genomic datasets that present both the issues explored in this paper, *i.e.,* that are both high-dimensional and class-imbalanced. It is a very challenging domain where the curse of dimensionality is a primary and critical concern since the number of features (genes) greatly overcomes the number of instances (biological samples), making the reduction of the data dimensionality an indispensable step. Indeed, the importance of identifying a reduced number of genes for medical diagnosis, while ensuring at the same time good predictive performance, has been widely highlighted in the literature (Saeys, Inza & Larranaga, 2007). In such a scenario, the contribution of this work is to comparatively evaluate the impact of feature selection when used alone, *i.e.,* without any strategy specifically designed to handle class imbalance, and when combined with cost-sensitive learning.

The results of our experiments, although not exhaustive, highlight the importance of jointly addressing high-dimensionality and class imbalance, giving useful insight into the benefits of a hybrid approach that relies on both feature selection and cost-sensitivity. Our study, in fact, shows that both feature selection and cost-sensitive classification can be greatly beneficial when used alone, but it is their combination that proves to be overall more convenient, leading to predictive models that can achieve good performance while exploiting only the most representative features of the domain at hand, with results that compare well with recent studies in the field. Furthermore, compared to other cost-sensitive approaches that rely on *ad hoc* algorithmic modifications, the strategies explored here have the advantage of being implementable with a variety of different methods, making them potentially suitable in a variety of scenarios.

The rest of the paper is organized as follows. In the next section (“Background concepts & Literature survey”), we provide background concepts on feature selection and imbalance learning techniques, with a brief survey of the main approaches and research lines presented in recent literature. The “Materials & Methods” section describes all the material and methods involved in our study, including the benchmarks, the feature selection methods, and the cost-sensitive learning strategies. The experimental study is presented in the “Experimental study” section with a summary and a discussion of the most interesting results. Finally, concluding remarks and future research directions are outlined in the last section (“Conclusions & Future Research Directions”).

## Background concepts & Literature survey

The background concepts relevant to our work are summarized in this section along three directions: *(i)* feature selection, *(ii)* imbalance learning methods, and *(iii)* hybrid approaches that leverage both feature selection and imbalance learning methods. Relevant proposals in the literature and open research problems are also outlined.

### Feature selection

A large corpus of literature has discussed the significant benefits of feature selection in high-dimensional learning tasks, *e.g.*, in terms of efficiency, generalization ability and interpretability of the induced models (Guyon & Elisseeff, 2003; Dessì & Pes, 2015a; Li et al., 2018). In fact, feature selection can remove irrelevant and noisy attributes, as well as redundant information, so making the learning algorithm focus on a reduced subset of predictive features.

Several selection methods have been proposed in the last years, which exploit different paradigms and heuristics (Kumar & Minz, 2014; Bolón-Canedo, Sánchez-Maroño & Alonso-Betanzos, 2015). Broadly, these methods can be categorized along two dimensions:

• *Evaluation of individual features or feature subsets*. Some selection techniques are designed to weight each single feature based on its correlation with the target class (ranking approach). Other methods exploit a proper search strategy (*e.g.*, a greedy search) to build different candidate subsets, whose quality is evaluated according to a proper criterion that tries to maximize the relevance of the selected features as well as to minimize their degree of redundancy (such a criterion may depend or not on the algorithm that will be used to induce the final model).
• *Interaction with the classifier. Filter* approaches carry out the selection process as a pre-processing step, only relying on the intrinsic characteristics of the data at hand, without any interaction with the classifier; *wrapper* methods use the classifier itself to evaluate different candidate solutions (*e.g.*, in terms of final predictive performance or considering both the performance and the number of selected features); *embedded* approaches leverage the internal capability of some learning algorithms to assess the relevance of the features for a given prediction task.

A significant amount of research has investigated the strengths and the limits of the different selection methods so far proposed, (*e.g.*, Saeys, Inza & Larranaga, 2007; Drotár, Gazda & Smékal, 2015; Bolón-Canedo et al., 2018; Bommert et al. , 2020). Hybrid and ensemble approaches, that properly combine different selection methods, have also been explored in the last years, with promising results in several application fields ((Dessì & Pes, 2015b; Almugren & Alshamlan, 2019; Bolón-Canedo & Alonso-Betanzos, 2019). However, it is not possible to find a feature selection technique that is best in all situations, and the choice of the most appropriate method for a given task remains often difficult (Oreski, Oreski & Klicek, 2017; Li et al., 2018).

Furthermore, little research has examined the effectiveness of the available feature selection algorithms in relation to the class imbalance problem (Haixiang et al., 2017). Indeed, when high-dimensionality and class imbalance coexist, the analysis may be intrinsically more complex due to an increased overlapping among the classes (Fu, Wu & Zong, 2020). In such a scenario, feature selection can potentially be quite helpful, although few studies have so far evaluated, in a comparative way, the behavior of different selection heuristics across imbalanced classification tasks (Zheng, Wu & Srihari, 2004; Cho et al., 2008; Wasikowski & Chen, 2010). Recently, some selection algorithms have also been modified to better deal with imbalanced data (Yin et al., 2013; Maldonado, Weber & Famili, 2014; Moayedikia et al., 2017), with positive results in dependence on the problem settings, but there is a lack of general methodological guidelines to fully exploit, in a synergic manner, both feature selection and imbalance learning methods (Zhang et al., 2019; Pes, 2020).

### Imbalance learning methods

Among the imbalance learning methods, some popular approaches act at the data level by modifying the class distribution in the original training data (He & Garcia, 2009; Branco, Torgo & Ribeiro, 2016). In particular, *under-sampling* techniques remove a fraction of instances of the majority class, either randomly or using some kind of informed strategy, while *over-sampling* techniques introduce new instances of the minority class, in order to reduce the level of class imbalance. In the first case, the major drawback is that some useful data can be discarded, with a reduction of the training set size (which may be problematic in small sample size domains). For oversampling, on the other hand, several authors agree that it can increase the risk of overfitting especially when exact copies of existing minority instances are made (Fernández et al., 2018a). A more sophisticated oversampling technique, the *SMOTE* approach, involves the introduction of new instances of the minority class by interpolating between existing minority instances that are close to each other (He & Garcia, 2009). This technique (with its extensions) has been successfully applied in a variety of domains, but its effectiveness in high-dimensional scenarios is still under debate and needs to be investigated in depth (Fernández et al., 2018b).

The ensemble classification paradigm has also been investigated as a potential solution to address class-imbalanced tasks (Galar et al., 2012; Zhao et al., 2021), but with limited applications on high-dimensional data (Lin & Chen, 2013), due to the intrinsically higher computational cost. A more efficient, and still effective, approach to deal with imbalanced data relies on the cost-sensitive paradigm (Ling & Sheng, 2010; López et al., 2013), where the different classification errors are penalized to a different extent in order to reduce the bias towards the majority class. The penalty terms, or costs, assigned to the errors are usually encoded in a cost matrix and are chosen in dependence on the characteristics of the domain at hand. Although there are many different ways of implementing cost-sensitive learning, the approaches discussed in the literature can be categorized into two main groups, *i.e.,* *(i)* ad hoc modification of existing learning algorithms and *(ii)* meta-learning approaches, independent of a specific classifier, that use the costs to act on the training instances or the classifier output (Ling & Sheng, 2010), as further discussed in the following section (“Materials & Methods”).

Interestingly, a number of empirical studies have shown that, in some application domains, cost-sensitive learning performs better than sampling methods (He & Garcia, 2009). Other authors have observed, with an extensive comparison between sampling methods and cost-sensitive techniques, that no approach always outperforms the other, the results being dependent on the intrinsic data characteristics (López et al., 2012). Despite the considerable amount of research in this field, the effectiveness of the different cost-sensitive techniques has yet to be comparatively explored in high-dimensionality problems, as most of the available studies focus on datasets with a relatively low number of features (López et al., 2013; Fernández et al., 2018a).

### Hybrid strategies

Recent literature has stressed the need to further investigate the combined effects of high dimensionality and class imbalance and to devise hybrid learning strategies that exploit, in a joint manner, both feature selection and imbalance learning methods. To this respect, a number of contributions have been made in the last years (Blagus & Lusa, 2013; Khoshgoftaar et al., 2014; Yin & Gai, 2015; Gao, Khoshgoftaar & Napolitano, 2015; Triguero et al., 2015; Shanab & Khoshgoftaar, 2018; Pes, 2020; Huang et al., 2021), mainly focused on studying suitable ways to integrate feature selection and sampling-based data balancing methods. Most of the results seem to indicate that using feature selection in conjunction with random under-sampling is generally better than with *SMOTE*, especially when the number of minority instances is quite low. On the other hand, no consensus exists on whether feature selection should be applied before or after data sampling, with results that depend on the specific problem at hand.

Another interesting, but less explored, area of research is the integration of feature selection and cost-sensitive learning. Indeed, some selection algorithms have been recently proposed that incorporate some kind of cost-sensitive correction, *e.g.*, using an ad hoc optimization function (Maldonado, Weber & Famili, 2014; Feng et al., 2020), but limited research has been done on cost-sensitive meta-learning approaches (Fernández et al., 2018a; Pes, 2021) that can be implemented in conjunction with different feature selection and classification algorithms (*e.g.*, acting on the instances’ weights). In this regard, methodological guidelines are still lacking, as well as comparative studies that investigate which strategy may be most suited (*e.g.*, introducing costs at the feature selection stage or at the model induction stage), and the impact of the adopted selection heuristic, based on the intrinsic properties of the data at hand (*e.g.*, instances-to-features ratio and degree of imbalance). This is the specific field where our work aims to give a contribution, as detailed in the rest of the paper.

### Materials & Methods

Focusing on a challenging application domain where the issues of high dimensionality and class imbalance may have a critical impact, this study evaluates the effectiveness of cost-sensitive learning strategies that incorporate a proper dimensionality reduction step, carried out through feature selection. All the materials and methods involved in our study are presented in what follows. Specifically, the first sub-section (“Genomic benchmarks”) describes the main characteristics of the genomic benchmarks used for the experiments. The second sub-section (“Feature selection methods”) illustrates the adopted ranking-based selection framework, with a description of the six selection algorithms chosen for the analysis. Finally, the third sub-section (“Integrating costs into the learning process”) discusses different ways to incorporate misclassification costs into the learning process, as well as different ways to combine them with feature selection.

### Genomic benchmarks

For our experiments, we chose three genomic benchmarks that encompass different levels of class imbalance. Specifically, the *DLBCL* dataset (Shipp et al., 2002) contains biological samples of diffuse large b-cell lymphoma (58 instances) and follicular lymphoma (19 instances), with only a moderate level of imbalance (*i.e.,* 25% of minority instances); each sample is described by the expression level of 7,129 genes, which leads to a very low instances-to-features ratio (*i.e.,* 0.01). In turn, the *Glioma* dataset (Nutt et al., 2003) has much more features (12,625 genes) than instances (50 biological samples), thus making the classification task quite challenging; in particular, in the binary version of the dataset here considered, the task is to discriminate between classic oligodendroglioma (14% of the instances) and other glioma types. Finally, the *Uterus* dataset (OpenML, 2021) has more instances, with a less critical—although still low—instances-to-features ratio (0.14); it contains indeed 1,545 biological samples, each described by the expression level of 10,935 genes. On the other hand, this benchmark also exhibits a more imbalanced data distribution, with only 8% of instances of the minority class (uterus cancer). For each of the considered datasets, the samples of the minority class have been modelled as positive and those of the majority class as negative, as usual practice in the imbalance learning field.

### Feature selection methods

Given the dimensionality of the data at hand, involving thousands of features, we exploited a ranking-based selection approach, which is indeed the primary choice in the presence of thousands of features (Saeys, Inza & Larranaga, 2007; Bolón-Canedo, Sánchez-Maroño & Alonso-Betanzos, 2015), as the size of the search space makes impractical the direct adoption of subset-oriented search strategies (they may still be very useful, however, to refine the selection process after a first, preliminary, dimensionality reduction).

Specifically, we considered both filter methods, that weight the features based on their correlation with the target class, using some statistical or entropic criterion, and embedded methods, that rely on the features’ weights derived by a suitable classifier. In both cases, the weights assigned to the features can be used to obtain a *ranked list* where the features appear in descending order of relevance (*i.e.,* from the most important to the least important): this list can be finally cut at a proper threshold point, to select a subset of predictive features to be used as input to the learning algorithm.

The six ranking methods chosen for the experiments are as follows:

• *Pearson’s correlation* (*CORR*), that evaluates the worth of each feature by measuring the extent to which its values are linearly correlated with the class (Tan et al., 2019): the higher the correlation, the more relevant the feature for the predictive task at hand. More in detail, the correlation between a feature *X* and the class attribute *Y* can be calculated using the expression:
\begin{eqnarray*}CORR(X,Y)= \frac{{\sigma }_{XY}}{{\sigma }_{X}{\sigma }_{Y}} \end{eqnarray*}
  where *σ*_*XY*_ is the covariance of *X* and *Y*, and *σ*_*X*_ and *σ*_*Y*_ are the standard deviations of *X* and *Y*, respectively.
• *Information Gain* (*IG*), that is able to capture more complex, not necessarily linear, dependencies among the class and the features. Specifically, *IG* relies on the information-theoretical concept of entropy (Witten et al., 2016): a weight is indeed computed for each feature by measuring how much the uncertainty in the class prediction decreases, *i.e.,* how much the class entropy decreases, when the value of the considered feature is known. By denoting as *H* the entropy function, we can therefore derive the *IG* value for a feature *X* as:
\begin{eqnarray*}IG(X)=H \left( Y \right) -H(Y{|}X) \end{eqnarray*}
  where *H(Y)* is the entropy of the class *Y* before observing *X*, while *H(Y—X)* is the conditional entropy of *Y* given *X* (Hall & Holmes, 2003).
• *Gain Ratio* (*GR*), that, similarly to *IG*, exploits the concept of entropy to assess the degree of correlation between a given feature and the class. However, *GR* tries to compensate for the *IG*’s bias toward features with more values by introducing a proper correction factor that considers how broadly the feature splits the data at hand (Witten et al., 2016). Specifically, such a correction is defined as

\begin{eqnarray*}SplitInfo \left( X \right) =-\sum _{i=1}^{r} \frac{{|}{X}_{i}{|}}{I} \cdot \log \nolimits 2 \frac{{|}{X}_{i}{|}}{I} \end{eqnarray*}

where —*X*_*i*_— is the number of training instances where *X* takes the value *X*_*i*_, *r* is the number of distinct values of *X*, and *I* is the total number of instances. The *GR* value for a feature *X* can then be obtained as:

\begin{eqnarray*}GR \left( X \right) =IG(X)/SplitInfo(X) \end{eqnarray*}

• *ReliefF (RF)*, that measures the worth of the features according to the extent to which they can discriminate between data instances that are near to each other (Urbanowicz et al., 2018). Iteratively, a sample instance is extracted from the dataset and its features’ values are compared to the corresponding values of the instance’s nearest neighbors (one, or more, for each class): the relevance of each feature is then measured based on the assumption that a predictive feature should have the same value for instances of the same class and different values for instances of different classes. More in detail, in the original two-class formulation, for each drawn sample instance *R*_*i*_ the algorithm finds its *nearest hit H* (nearest neighbor from the same class) and its *nearest miss M* (nearest neighbor from the opposite class). Starting from a null weight for the feature *X* under evaluation, *i.e.,* *W(X)* =* 0*, such a weight is iteratively updated as follows:
\begin{eqnarray*}W(X):=W(X)-diff(X,{R}_{i},H)/m+diff(X,{R}_{i},M)/m \end{eqnarray*}
  where *m* is the number of randomly drawn sample instances (it can also coincide with the total number of instances, as in our implementation) and *diff* is a function that computes the difference between the value of *X* for two instances: the difference computed for *R*_*i*_ and *H*, *diff(X, R*_*i*_*,H)*, makes *W(X)* lower, while the difference computed for *R*_*i*_ and *M*, *diff(X, R*_*i*_*,M)*, increases it. Such a binary formulation can be extended to also handle multi-class and noisy data (Robnik-Sikonja & Kononenko, 2003).
• *SVM-AW*, that leverages a linear *Support Vector Machine* (*SVM*) classifier to assign a weight to each feature. Indeed, the *SVM* algorithm looks for an optimal hyperplane as a decision function to separate the instances in the feature space:
\begin{eqnarray*}f \left( \mathbf{x} \right) =\mathbf{w}\cdot \mathbf{x}+b \end{eqnarray*}
  where ***x*** is an instance vector in the *N*-dimensional space of input features, ***w*** is a weight vector, and *b* is a bias constant. In this function, each feature (*i.e.,* space dimension) is assigned a weight that can be interpreted as the feature’s contribution to the multivariate decision of the classifier. Such a weight can be assumed, in absolute value, as a measure of the strength of the feature (Rakotomamonjy, 2003).
• *SVM-RFE*, that, similarly to *SVM-AW*, relies on the features’ weights derived by a linear *SVM* classifier. However, the *SVM-RFE* approach involves a recursive feature elimination strategy that iteratively removes a given percentage of the least predictive features (those with the lowest weights) and repeats the hyperplane function induction on the remaining features, which are hence reweighted accordingly (Guyon et al., 2002; Rakotomamonjy, 2003). The computational complexity of the method is strongly influenced by the percentage *p* of features removed at each iteration: when *p* = 100%, *SVM-RFE* reduces to *SVM-AW* as all the features are ranked in one step; when *p* <100%, the overall ranking of features is constructed in an iterative way, at a higher computation cost (the lower *p*, the greater the number of iterations).For our study, the parameter *p* was set as 50%, in order to contain the computational cost of the method.

As summarized above, the considered ranking methods exploit quite different heuristics. Indeed, *CORR*, *IG*, *GR*, and *RF* do not leverage any classifier and can be thus categorized as filters, while *SVM-AW* and *SVM-RFE* are two popular representatives of the embedded selection techniques (Saeys, Inza & Larranaga, 2007). On the other hand, from a different perspective, these methods can be distinguished into univariate (*CORR*, *IG*, and *GR*) and multivariate (*RF*, *SVM-AW*, and *SVM-RFE*) approaches: the first group assesses the relevance of each feature independently of the other features, while the methods in the second group can capture, to some extent, the inter-dependencies among the features (indeed, the instances’ position in the attribute space contributes to determining, in a multivariate way, both the *RF*’s ranks and the *SVM*’s weights). Although widely employed in different application contexts, the above selection methods are still to be exhaustively evaluated in connection with the class imbalance problem, especially in the presence of low instances-to-features ratios.

### Integrating costs into the learning process

As discussed previously in the “Background concepts & Literature survey” section, the cost-sensitive paradigm has been largely explored in the context of imbalanced data analysis (He & Garcia, 2009; López et al., 2013), but most of the reported applications refer to low-dimensional datasets. Basically, this paradigm involves taking misclassification costs into consideration, in order to induce models that minimize the total cost of the errors rather than the number of errors, as traditional classifiers typically do. Indeed, in several real-world scenarios, the incorrect classification of a rare instance (*e.g.*, a rare disease or an illegal transaction) may have more costly implications and consequences, which makes it crucial to reduce such kind of errors as much as possible.

Although a number of learning algorithms have been designed to be cost-sensitive in themselves (Ling & Sheng, 2010; Fernández et al., 2018a), our focus here is on a methodological framework that can be adopted to convert a generic learner into a cost-sensitive one. Specifically, a *cost matrix* can be defined (*e.g.*, based on domain knowledge) that expresses the cost *C(i,j)* of classifying an instance of class *i* as an instance of class *j*. Assuming a binary scenario, with a minority (*positive*) and a majority (*negative*) class, a *false negative* error (*i.e.,* a positive instance incorrectly classified as a negative one) is given higher cost than a *false positive* error (*i.e.,* a negative instance incorrectly classified as a positive one), while the costs for the correct predictions are typically set to zero (or to some negative value, which can be interpreted as a “reward” that reduces the overall cost of the model). Hence, for a given cost matrix, an instance *x* should be classified into the class *j* that has the *minimum expected cost*, defined as:

\begin{eqnarray*}R \left( j{|}x \right) =\sum _{i}P(i{|}x)\cdot C(i,j) \end{eqnarray*}

where *P(i—x)* is the probability estimation of classifying an instance *x* into class *i*. It can be shown that a proper probability threshold *pth* can be derived to classify an instance into positive if *P(+—x) >* =* pth* (Ling & Sheng, 2010), where:

\begin{eqnarray*}pth= \frac{C(-,+)}{ \left( C-,+ \right) +C(+,-)} . \end{eqnarray*}

**Figure 1:**
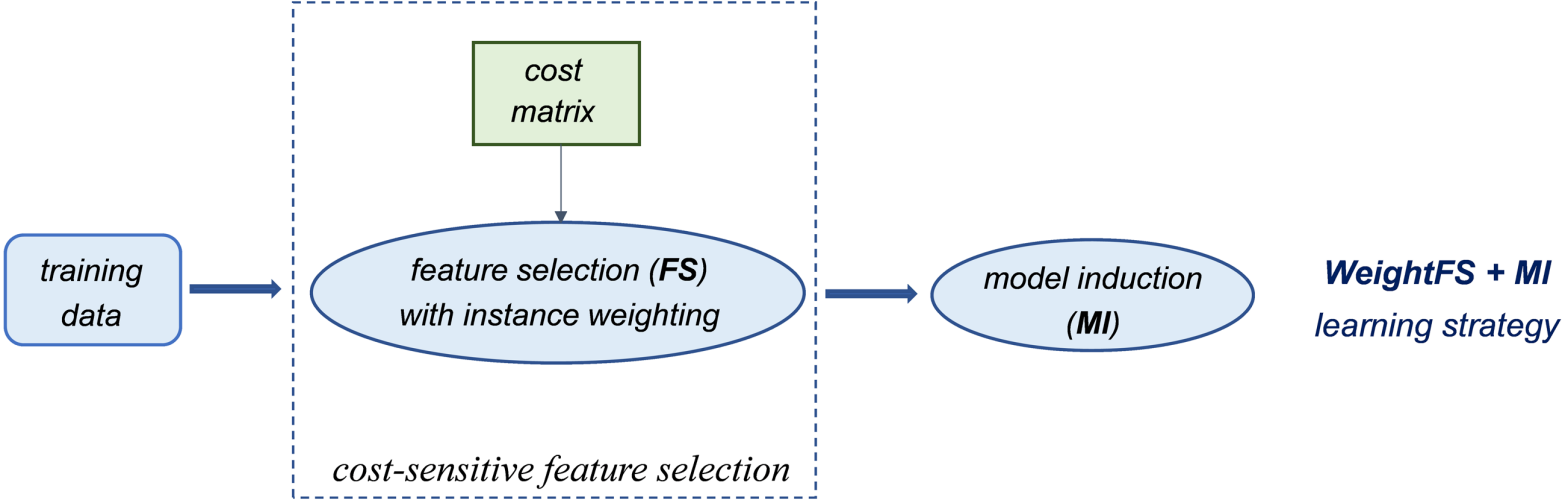
Introducing cost-sensitivity at the feature selection stage.

**Figure 2:**
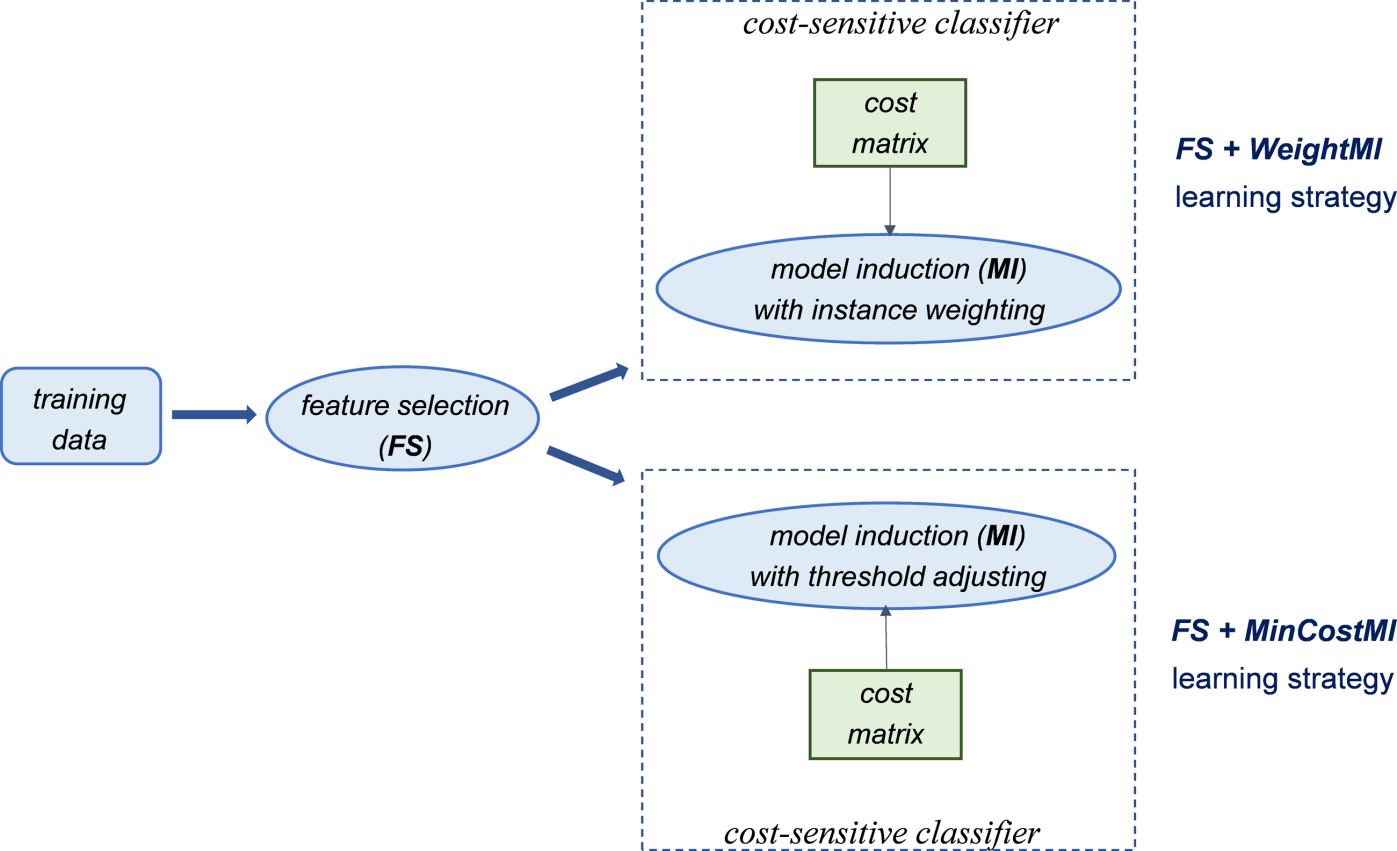
Introducing cost-sensitivity at the model induction stage.

Alternatively, cost-sensitivity can be achieved by weighting the instances of each class according to their misclassification costs, without acting on the classifier threshold. This means that higher weights are assigned to the instances of the minority class (which has a higher misclassification cost). Such a weighting mechanism can be used at different stages of the learning process, which may lead to quite different outcomes, as discussed in the following section. Specifically, since our methodological approach relies on integrating both cost-sensitivity and feature selection into the learning process, we consider and compare different strategies that are schematized in Figs. 1 and 2.

Essentially, the first strategy (*WeightFS+MI*) consists in reweighting the instances at the feature selection (*FS*) stage, according to the given cost matrix (Fig. 1). This way, the feature selection itself is made cost-sensitive, without any action at the model induction (*MI*) stage. In contrast, the other two strategies make the classifier cost-sensitive (Fig. 2), either with an instance weighting mechanism (*FS+WeightMI* strategy) or acting on the probability threshold to minimize the expected cost (*FS+MinCostMI* strategy). The effectiveness of such learning strategies is evaluated in this study for different cost matrices, in order to investigate the optimal cost settings based on the intrinsic data characteristics, as discussed in what follows.

### Experimental study

In this section, we first present the specific settings of our experiments, along with the metrics employed for performance evaluation (“Experimental settings & Evaluation metrics”). Next, the main experimental results are illustrated and discussed (“Results & Discussion”).

### Experimental settings & evaluation metrics

For each of the benchmarks described above, the cost-sensitive learning strategies shown in Fig. 1 and Fig. 2 have been evaluated in conjunction with different feature selection methods (*CORR*, *IG*, *GR*, *RF*, *SVM-AW*, *SVM-RFE*), as well as for different levels of data reduction, *i.e.,* selecting feature subsets of different sizes. As a learning algorithm for model induction, we exploited the *Random Forest* classifier (Breiman, 2001), which has proven to be a suitable choice in the genomic domain here considered (Chen & Ishwaran, 2012; Pes, 2020), as well as across different application contexts (Rokach, 2016), even in the presence of imbalanced data distributions (Khoshgoftaar, Golawala & Van Hulse, 2007; Bartoletti, Pes & Serusi, 2018; Walker & Hamilton, 2019; Chicco & Oneto, 2021). Specifically, we relied on commonly adopted settings which involve a forest of 100 trees, each built choosing, at the splitting stage, the best attribute among a number *log*_2_*(n)+1* of random features (where *n* is the dataset dimensionality). For the *Random Forest* classifier, as well as for the six considered selection methods, we exploited the implementations provided by the *WEKA* machine learning workbench (Weka, 2021), which also provides proper meta-functions supporting cost-sensitive learning.

More in detail, the settings adopted for the *Random Forest* classifier correspond to the default parameters in the *WEKA* library. The *WEKA CorrelationAttributeEval*, *InfoGainAttributeEval*, *GainRatioAttributeEval*, and *ReliefFAttributeEval* functions, with their default settings, have been used to implement the filter methods *CORR*, *IG*, *GR*, and *RF*, respectively. For the embedded approaches *SVM-AW* and *SVM-RFE*, we exploited the *SVMAttributeEval* function, by setting the percentage of features to eliminate per iteration as 100% and 50% respectively. Each of these attribute evaluation functions has been coupled with the *Ranker* search method that allows selecting the desired number of top-ranked features. Further, to introduce cost-sensitivity at the feature selection stage, we relied on the *CostSensitiveAttributeEval* meta-function that can wrap any of the adopted selectors and make it cost-sensitive based on a given cost matrix. Similarly, the *CostSensitiveClassifier* meta-function has been used to introduce cost-sensitivity at the model induction stage, acting both on the instances’ weights or on the probability threshold of the classifier.

As regards performance evaluation, we considered proper measures that can reliably estimate the model capability of discriminating among imbalanced classes (Luque et al., 2019). In particular, the *Matthews Correlation Coefficient* (*MCC*) expresses the degree of correlation between the observed and predicted classifications:

\begin{eqnarray*}MCC= \frac{TP\cdot TN-FP\cdot FN}{\sqrt{(TP+FP)(TP+FN)(TN+FP)(TN+FN)}} \end{eqnarray*}

where, according to the commonly adopted notation, *TP* is the number of *true positives*, *i.e.,* the actual positives that are correctly classified as positives; *TN* is the number of *true negatives*, *i.e.,* the actual negatives that are correctly classified as negatives; *FP* is the number of *false positives*, *i.e.,* the actual negatives that are wrongly classified as positives; *FN* is the number of *false negatives*, *i.e.,* the actual positives that are wrongly classified as negatives. As highlighted in recent literature, the *MCC* measure turns out to be very trustworthy on imbalanced datasets (Chicco, 2017; Chicco, Warrens & Jurman, 2021).

Another performance metric widely adopted in the context of imbalance learning is the *G-mean* (Branco, Torgo & Ribeiro, 2016). It is defined as the geometric mean between the fraction of positive instances classified correctly (*TP rate* or *sensitivity*) and the fraction of negative instances classified correctly (*TN rate* or *specificity*):

\begin{eqnarray*}G-mean=\sqrt{sensitivity\cdot specificity}. \end{eqnarray*}

Such a mean takes into account the capability of the model of discriminating each single class, providing a useful trade-off between different types of errors, *i.e.,* the false negatives (that affect the sensitivity, namely *TP/(TP+FN)*) and the false positives (that affect the specificity, namely *TN/(TN+FP)*).

A different way to account for both false positives and false negatives is to jointly consider the *sensitivity* and the *precision*, which expresses the fraction of instances that are actually positive in the group the model has classified as positive (namely *TP/(TP+FP)*). In particular, the well-known *F-measure* is defined as the harmonic mean between the sensitivity and the precision:

\begin{eqnarray*}F-measure= \frac{2\cdot sensitivity\cdot precision}{sensitivity+precision} . \end{eqnarray*}

By using multiple performance measures (*MCC*, *G-mean*, *F-measure*), we aim to obtain a more reliable insight into the effectiveness of the considered learning strategies. To estimate such measures in a robust way, we considered their average value across different model training-testing runs. Specifically, for each of the considered datasets, we performed a *5-fold stratified cross-validation*, repeated 4 times, as in similar studies dealing with high-dimensional and imbalanced data, *e.g.*, (Khoshgoftaar et al., 2014; Shanab & Khoshgoftaar, 2018). Each run of 5-fold cross-validation leads to 5 different partitions of the original data into training and test set (respectively 80% and 20% of the records). By repeating the cross-validation four times, we obtained 20 different partitions with the same percentage of training and test data. This is somewhat similar to a repeated holdout protocol where different training/test sets are drawn from the original dataset: the overall learning process (feature selection and model induction) has been repeated 20 times (for each learning strategy and each specific setting), each time using a different training set for model induction and the corresponding test set for performance evaluation. Finally, all the evaluation metrics have been averaged across the 20 runs, to reduce any possible bias due to a specific data partitioning.

## Results & discussion

The first step of our experimental study involves evaluating the extent to which the considered selection methods (see sub-section “Feature selection methods”) are useful in mitigating the adverse effects of class imbalance. Indeed, although such methods have been widely employed across several application contexts, their effectiveness in high-dimensional and imbalanced tasks is yet to be investigated in depth.

**Figure 3:**
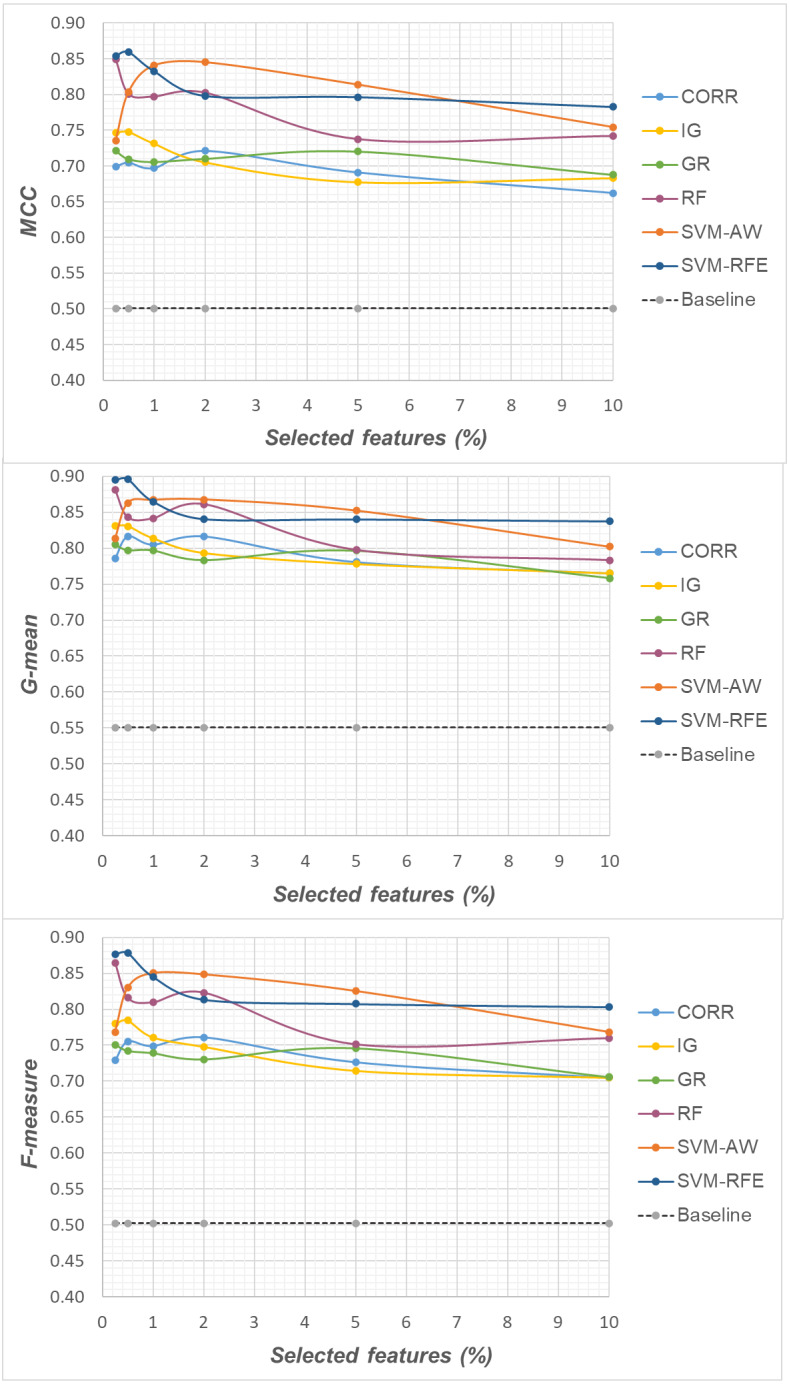
*DLBCL* dataset: *MCC*, *G-mean* and *F-measure* performance in conjunction with different selection methods (*CORR*, *IG*, *GR*, *RF*, *SVM-AW*, *SVM-RFE*), for different percentages of selected features.

**Figure 4:**
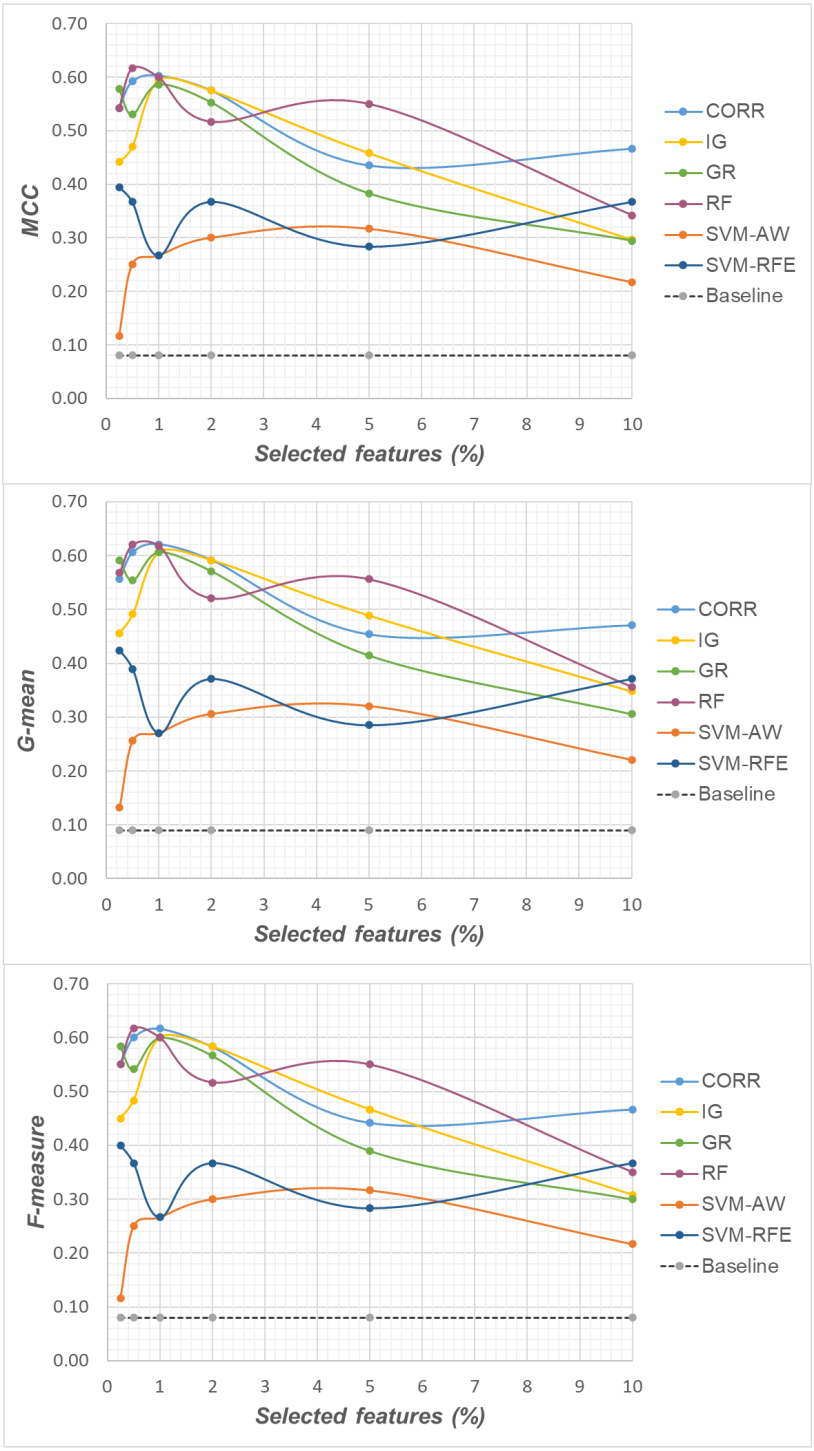
*Glioma* dataset: *MCC*, *G-mean* and *F-measure* performance in conjunction with different selection methods (*CORR*, *IG*, *GR*, *RF*, *SVM-AW*, *SVM-RFE*), for different percentages of selected features.

**Figure 5:**
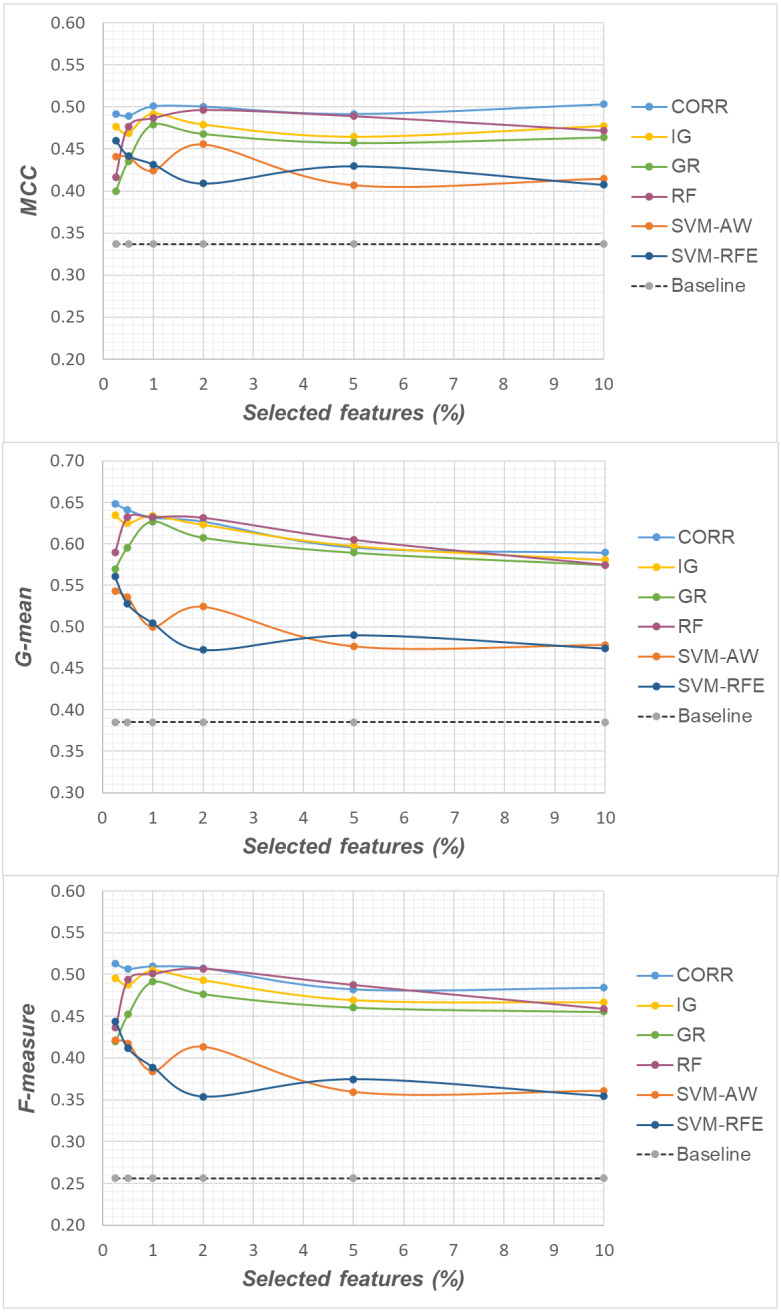
*Uterus* dataset: *MCC*, *G-mean* and *F-measure* performance in conjunction with different selection methods (*CORR*, *IG*, *GR*, *RF*, *SVM-AW*, *SVM-RFE*), for different percentages of selected features.

Specifically, as the employed techniques act by ranking the features according to their degree of relevance, different threshold values have been considered to cut the resulting ranked lists, in order to obtain feature subsets of different sizes. This allowed us to explore the impact of different levels of dimensionality reduction on the performance metrics mentioned above (see previous sub-section). The results obtained on the *DLBCL*, *Glioma* and *Uterus* datasets are shown in Figs. 3, 4, and 5 respectively. In each figure, we show the predictive performance of the *Random Forest* classifier, in terms of *MCC*, *G-mean* and *F-measure*, when used alone (baseline model), *i.e.,* without any dimensionality reduction, as well as when used in conjunction with the different selection methods (*CORR*, *IG, GR*, *RF*, *SVM-AW*, *SVM-RFE*); note that the baseline model is represented by a dashed line in the figures.

A first point to highlight is that the feature selection process seems to be effective in improving the classifier performance, either in the presence of a moderate imbalance level (*DLBCL* dataset) as well as for more skewed class distributions (*Glioma* and *Uterus* datasets). Indeed, for each of the employed metrics, *i.e.,* the *MCC*, the *G-mean* and the *F-measure*, the baseline values are quite low, especially for the *Glioma* dataset that has the lowest instance-to-features ratio (0.004), with only 7 positive instances in total. As we can see in the figures, when the data dimensionality is properly reduced, a considerable improvement in performance can be achieved. The statistical significance of such an improvement was assessed by applying the *Wilcoxon signed-rank test* (Demšar, 2006), which is a non-parametric alternative to the paired *t*-test for comparing two classifiers over different data samples. Specifically, for each performance measure, we compared the outcome obtained with and without feature selection (*i.e.,* the baseline model). Fixing the level of dimensionality reduction, *i.e.,* the percentage of selected features, this comparison was carried out for each of the six considered selection methods (*CORR, IG, GR, RF, SVM-AW, SVM-RFE*), leading to six separate comparisons against the baseline. To address the issue of multiple hypothesis testing, the *Holm-Bonferroni* correction was applied that involves ordering the *p*-values from most significant to the least significant: *p*_1_, *p*_2_, …, *p*_*k*_ (where *k* is the number of the hypotheses). Then, if *p*_1_ is below *α/k*, the corresponding null hypothesis (no statistical difference) is rejected and we are allowed to compare the second *p*_2_ with *α/(k-1)*; if the second hypothesis is rejected too, the test proceeds with the third, and so on. In our setting, *α* = 0.05 and *k* = 6. The results of this analysis are summarized in Table 1 considering, as an example, the models obtained by selecting 2% of the original features. The performance values that turned out to be significantly different from the baseline are marked in bold, with the corresponding *p*-values in brackets.

**Table 1:** Comparison of the classification performance achieved with and without feature selection, for (a) the DLBCL dataset, (b) the Glioma dataset and (c) the Uterus dataset. The comparison is carried out for each of the considered selection methods (by retaining 2% of the original features). The values in bold turned out to be significantly different from the baseline according to the Wilcoxon signed-rank test with Holm-Bonferroni correction (*p*-values in brackets).

**(a)**	**Baseline**	**CORR**	**IG**	**GR**	**RF**	**SVM-AW**	**SVM-RFE**
*MCC*	0.50	**0.72**	**0.71**	**0.71**	**0.80**	**0.85**	**0.80**
		*(0.0024)*	*(0.0085)*	*(0.0035)*	*(0.0006)*	*(0.0008)*	*(0.0008)*
*G-mean*	0.55	**0.82**	**0.79**	**0.78**	**0.86**	**0.87**	**0.84**
		*(0.0010)*	*(0.0017)*	*(0.0010)*	*(0.0006)*	*(0.0007)*	*(0.0008)*
*F-measure*	0.5	**0.76**	**0.75**	**0.73**	**0.82**	**0.85**	**0.81**
		*(0.0019)*	*(0.0038)*	*(0.0023)*	*(0.0009)*	*(0.0007)*	*(0.0008)*
**(b)**	**Baseline**	**CORR**	**IG**	**GR**	**RF**	**SVM-AW**	**SVM-RFE**
*MCC*	0.08	**0.58**	**0.58**	**0.55**	**0.52**	**0.30**	**0.37**
		*(0.0012)*	*(0.0012)*	*(0.0017)*	*(0.0024)*	*(0.0267)*	*(0.0131)*
*G-mean*	0.09	**0.59**	**0.59**	**0.57**	**0.52**	**0.31**	**0.37**
		*(0.0012)*	*(0.0012)*	*(0.0014)*	*(0.0024)*	*(0.0267)*	*(0.0131)*
*F-measure*	0.08	**0.58**	**0.58**	**0.57**	**0.52**	**0.30**	**0.37**
		*(0.0012)*	*(0.0012)*	*(0.0014)*	*(0.0024)*	*(0.0267)*	*(0.0131)*
**(c)**	**Baseline**	**CORR**	**IG**	**GR**	**RF**	**SVM-AW**	**SVM-RFE**
*MCC*	0.34	**0.50**	**0.48**	**0.47**	**0.50**	**0.46**	**0.41**
		*(<0.0001)*	*(<0.0001)*	*(<0.0001)*	*(<0.0001)*	*(<0.0001)*	*(0.0004)*
*G-mean*	0.38	**0.63**	**0.62**	**0.61**	**0.63**	**0.52**	**0.47**
		*(<0.0001)*	*(<0.0001)*	*(<0.0001)*	*(<0.0001)*	*(<0.0001)*	*(0.0002)*
*F-measure*	0.26	**0.51**	**0.49**	**0.48**	**0.51**	**0.41**	**0.35**
		*(<0.0001)*	*(<0.0001)*	*(<0.0001)*	*(<0.0001)*	*(<0.0001)*	*(0.0002)*

Based on Table 1, as well as the curves in Figs. 3–5, some interesting insight can be derived by comparing the performance of the different selection methods across the three examined benchmarks. In particular, the *SVM*-based methods, *i.e.,* the multivariate *SVM-AW* and *SVM-RFE*, seem to suffer to a greater extent as the degree of imbalance increases (Figs. 4 and 5), albeit achieving the best results on the *DLBCL* dataset (Fig. 3). On the other hand, the other multivariate method, *RF*, exhibits a quite satisfactory behavior across different levels of imbalance and instances-to-features ratios. As well, the univariate methods (*CORR*, *IG*, *GR*), despite showing slightly worse performance on the *DLBCL* dataset, seem to be a suitable option on the most imbalanced benchmarks, at least for small percentages of selected features. Furthermore, they have the advantage of being computationally less expensive than the multivariate approaches.

Overall, no single method turns out to be better across the different settings explored in Figs. 3–5 but, irrespective of the chosen selection approach, the dimensionality reduction step has proven to be beneficial in this first phase of our analysis, besides having undoubtful advantages in terms of knowledge discovery (as it can identify the most predictive features for the considered domain). As a further and fundamental step of our experiments, we investigated whether, and under which conditions, the use of hybrid learning strategies that involve both feature selection and cost-sensitivity can be further beneficial. Specifically, as schematized in Figs. 1 and 2, we considered both making the feature selection itself cost-sensitive (*WeightFS+MI* strategy) as well as introducing costs at the model induction stage (*FS+WeightMI and FS+MinCostMI* strategies). For each of these strategies, the evaluation has been performed in conjunction with different selection methods and different percentages of selected features.

**Figure 6:**
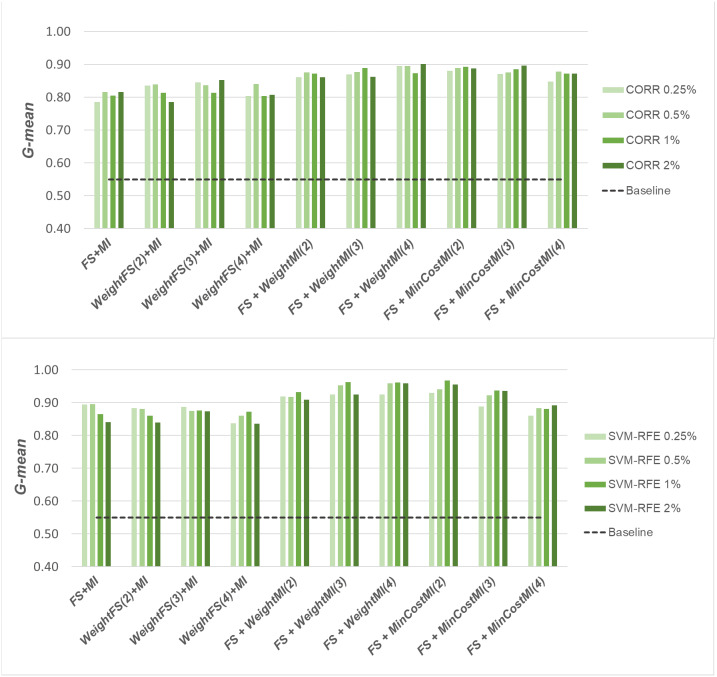
*DLBCL* dataset: *G-mean* performance achieved with different learning strategies, in conjunction with the *CORR* method and the *SVM-RFE* method, for different percentages of selected features.

**Figure 7:**
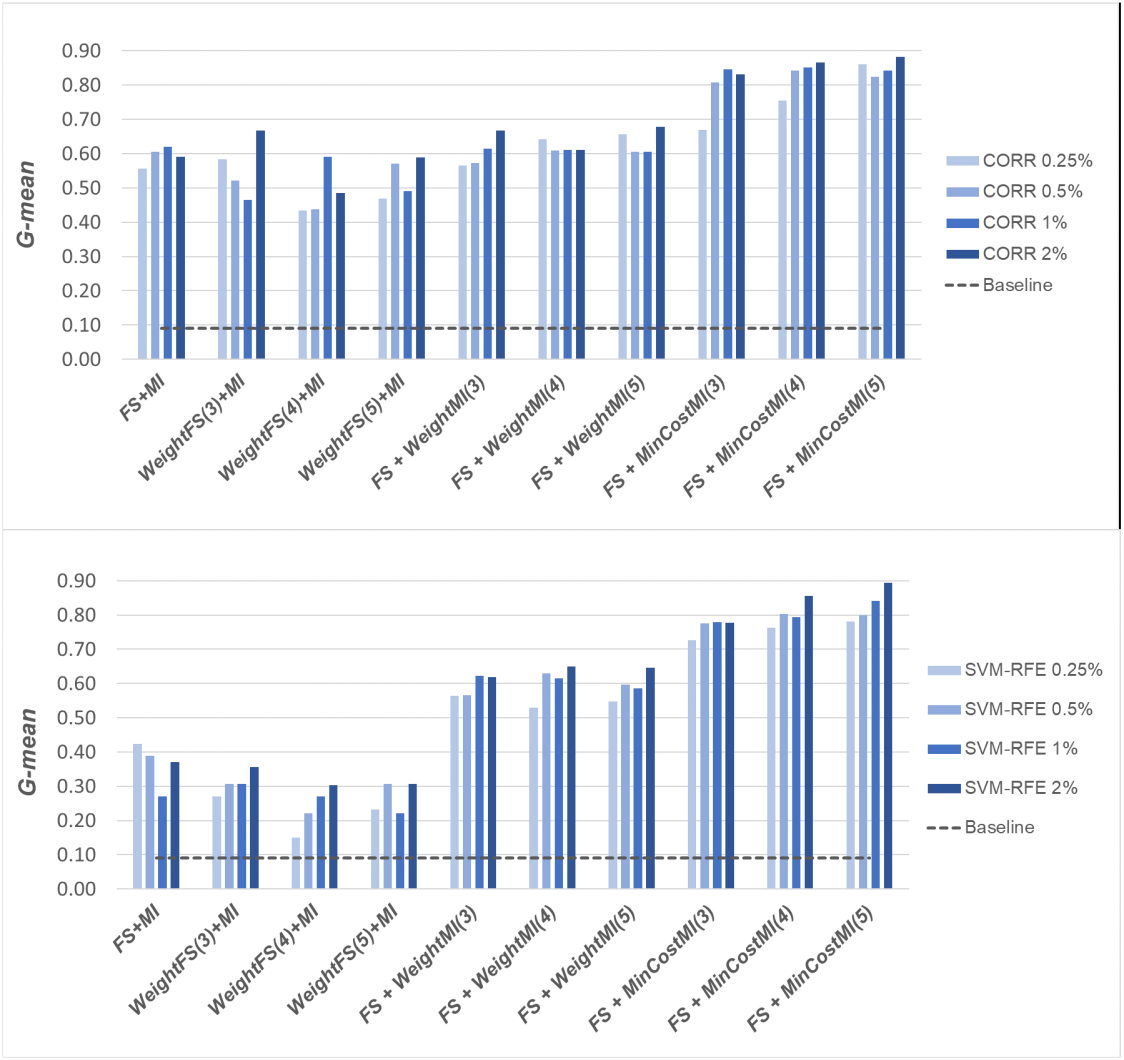
*Glioma* dataset: *G-mean* performance achieved with different learning strategies, in conjunction with the *CORR* method and the *SVM-RFE* method, for different percentages of selected features.

**Figure 8:**
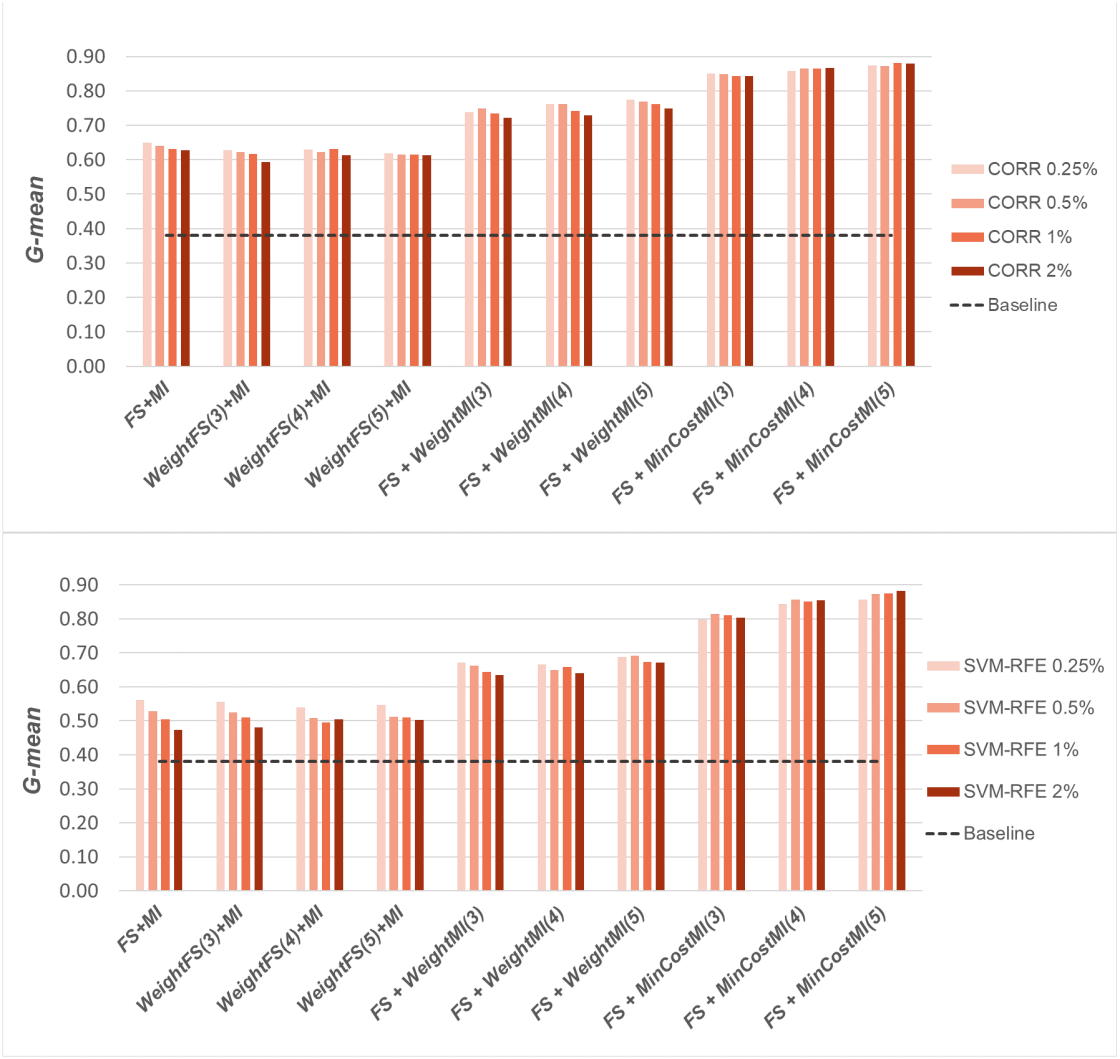
*Uterus* dataset: *G-mean* performance achieved with different learning strategies, in conjunction with the *CORR* method and the *SVM-RFE* method, for different percentages of selected features.

A first comparative view of the results is shown in Figs. 6–8 that refer to *DLBCL*, *Glioma* and *Uterus* datasets respectively. For the sake of space and readability, we only show here the *G-mean* values obtained in conjunction with the *CORR* method, as representative of the univariate approach, and the *SVM-RFE* method, as representative of the multivariate approach; for both the methods, we focus on small percentages of selected features, from 0.25% to 2%, that are usually more interesting in practical applications. In each figure, the results obtained by simply carrying out the feature selection before inducing the model (*FS + MI* approach) are compared with those achieved with the three considered hybrid strategies, *i.e., WeightFS(c)+MI*, *FS+WeightMI(c)* and *FS+MinCostMI(c)*, where *c* is the cost assigned to the false negatives (*i.e.,* the positive/minority instances classified incorrectly). As we can see, different values have been explored for *c* (three settings that have proven interesting are shown in each figure), while a (fixed) unitary cost has been assigned to the false positives (*i.e.,* the negative/majority instances classified incorrectly), with no cost for the correct predictions. The performance of the baseline model, without any dimensionality reduction or cost-sensitive correction, has also been shown in the figures (dashed line).

When comparing the outcome of the different learning strategies, we can observe that using an instance weighting mechanism at the feature selection stage (*WeightFS+MI*) is not advantageous compared to the simpler *FS + MI* strategy that is actually able to improve the baseline performance, as also observed previously in Figs. 3–5, without any cost-sensitive correction. On the other hand, introducing costs at the model induction stage, after reducing the data dimensionality, can be strongly beneficial especially in the presence of a high level of class imbalance. In all the considered benchmarks, in fact, the hybrid strategies *FS+WeightMI* and *FS+MinCostMI* have proven to be more convenient than using feature selection alone (*FS + MI*). In particular, although weighting instances at the model induction stage (*FS+WeightMI*) turns out to be a good option on the *DLBCL* dataset (Fig. 6), the *FS+MinCostMI* approach seems to be overall more convenient across the different settings here explored, with a significant improvement of the prediction performance on the most imbalanced datasets (Figs. 7 and 8). As regards the choice of the *c* parameter, *i.e.,* the cost assigned to the misclassified minority instances, we can see that it should be increased with the increase of the imbalance level; however, values higher than 5 were not found convenient in our case study, due to the greater amount of false positives.

Once again, we applied the Wilcoxon signed-rank test, with Holm-Bonferroni correction, to assess whether the differences observed in Figs. 6–8 are statistically significant. In particular, we compared the *G-mean* performance achieved using the hybrid strategies *FS+WeightMI* and *FS+MinCostMI* with that achieved using feature selection alone (*FS + MI*). For each strategy, we considered three cost settings (corresponding to different values of the *c* parameter), leading to three distinct comparisons against the *FS + MI* approach. The results of this analysis (considering *α* = 0.05 and *k* = 3) are summarized in Table 2, for two different percentages of selected features (*i.e.,* 0.25% and 2%). We marked in bold the performance values that were found to be significantly different from those obtained with feature selection alone, with the corresponding *p*-values in brackets. As we can see, the hybrid strategies were confirmed to be overall more convenient than the *FS + MI* approach, which in turn was found to be better than the baseline classifier (Table 1). More in detail, the significance of the performance improvements achieved with the hybrid approach may depend on how the feature selection is carried out (adopted selection method and level of dimensionality reduction), besides the intrinsic data characteristics. Indeed, in the least imbalanced dataset, *i.e.,* DLBCL (Table 2A), feature selection alone may lead to quite satisfactory results, in dependence on the adopted settings, while the hybrid strategies always perform significantly better, irrespective of the adopted settings, in the *Uterus* dataset (Table 2C), which is the most imbalanced.

**Table 2:** *G-mean* measured using a hybrid learning strategy (*FS* + *WeightMI* or *FS* + *MinCostMI*) or feature selection alone (*FS* + *MI*), for (a) the *DLBCL* dataset, (b) the *Glioma* dataset and (c) the *Uterus* dataset. The comparison is carried out, separately for the *FS* + *WeightMI* and *FS* + *MinCostMI* strategies, using *CORR* and *SVM*-*RFE* as selection methods (by retaining 0.25% and 2% of the original features). The values in bold turned out to be significantly different from those obtained with the *FS* + *MI* approach according to the Wilcoxon signed-rank test with Holm-Bonferroni correction (*p*-values in brackets).

	**(a)**	***FS+ WeightMI(c)***			***FS+MinCostMI(c)***		
	***FS+MI***	**c = 2**	**c = 3**	**c = 4**	**c = 2**	**c = 3**	**c = 4**
**CORR 0.25%**	0.79	**0.86** *(0.0119)*	**0.87** *(0.0053)*	**0.89** *(0.0010)*	**0.88** *(0.0029)*	**0.87** *(0.0059)*	**0.85** *(0.0243)*
**CORR 2%**	0.82	0.86 *(0.1427)*	0.86 *(0.1427)*	**0.90** *(0.0021)*	**0.89** *(0.0048)*	**0.90** *(0.0093)*	0.87 *(0.0515)*
**SVM-RFE 0.25%**	0.90	0.92 *(0.2204)*	0.92 *(0.0774)*	0.93 *(0.1105)*	0.93 *(0.0659)*	0.89 *(0.2797)*	0.86 *(0.0779)*
**SVM-RFE 2%**	0.84	**0.91** *(0.0091)*	**0.93** *(0.0121)*	**0.96** *(0.0024)*	**0.96** *(0.0007)*	**0.94** *(0.0098)*	0.89 *(0.1436)*
	**(b)**	***FS+ WeightMI(c)***			***FS+MinCostM/(c)***		
	***FS+MI***	**c = 3**	**c = 4**	**c = 5**	**c = 3**	**c = 4**	**c = 5**
**CORR 0.25%**	0.56	0.56 *(1.00)*	0.64 *(0.2919)*	0.66 *(0.1404)*	0.67 *(0.1099)*	0.75 *(0.0324)*	**0.86** *(0.0141)*
**CORR 2%**	0.59	0.67 *(0.1714)*	0.61 *(0.3759)*	0.68 *(0.0700)*	**0.83** *(0.0178)*	**0.87** *(0.0143)*	**0.88** *(0.0113)*
**SVM-RFE 0.25%**	0.42	0.56 *(0.0284)*	0.53 *(0.1030)*	0.55 *(0.1342)*	**0.73** *(0.0041)*	**0.76** *(0.0024)*	**0.78** *(0.0017)*
**SVM-RFE 2%**	0.37	**0.62** *(0.0157)*	**0.65** *(0.0100)*	**0.65** *(0.0062)*	**0.78** *(0.0019)*	**0.86** *(0.0009)*	**0.89** *(0.0008)*
	**(c)**	***FS+ WeightMI(c)***			***FS+MinCostMI(c)***		
	***FS+MI***	**c = 3**	**c = 4**	**c = 5**	**c = 3**	**c = 4**	**c = 5**
**CORR 0.25%**	0.65	**0.74** *(<0.0001)*	**0.76** *(<0.0001)*	**0.77** *(<0.0001)*	**0.85** *(<0.0001)*	**0.86** *(<0.0001)*	**0.87** *(<0.0001)*
**CORR 2%**	0.63	**0.72** *(<0.0001)*	**0.73** *(<0.0001)*	**0.75** *(<0.0001)*	**0.84** *(<0.0001)*	**0.87** *(<0.0001)*	**0.88** *(<0.0001)*
**SVM-RFE 0.25%**	0.56	**0.67** *(<0.0001)*	**0.66** *(<0.0002)*	**0.69** *(<0.0001)*	**0.80** *(<0.0001)*	**0.84** *(<0.0001)*	**0.86** *(<0.0001)*
**SVM-RFE 2%**	0.47	**0.63** *(<0.0001)*	**0.64** *(<0.0001)*	**0.67** *(<0.0001)*	**0.80** *(<0.0001)*	**0.86** *(<0.0001)*	**0.88** *(<0.0001)*

**Figure 9:**
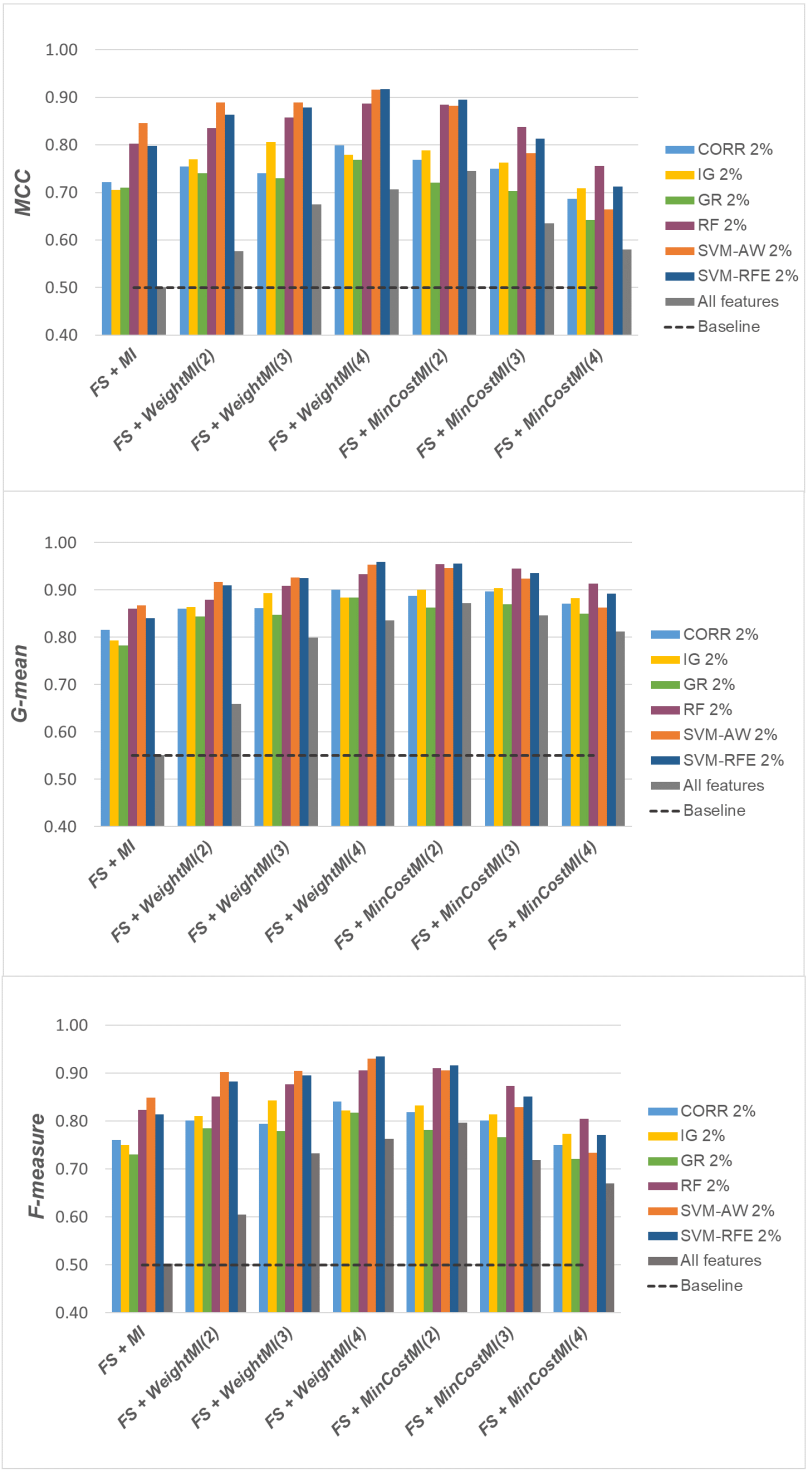
*DLBCL* dataset: *MCC*, *G-mean* and *F-measure* performance achieved with different learning strategies, in conjunction with the six considered selection methods.

**Figure 10:**
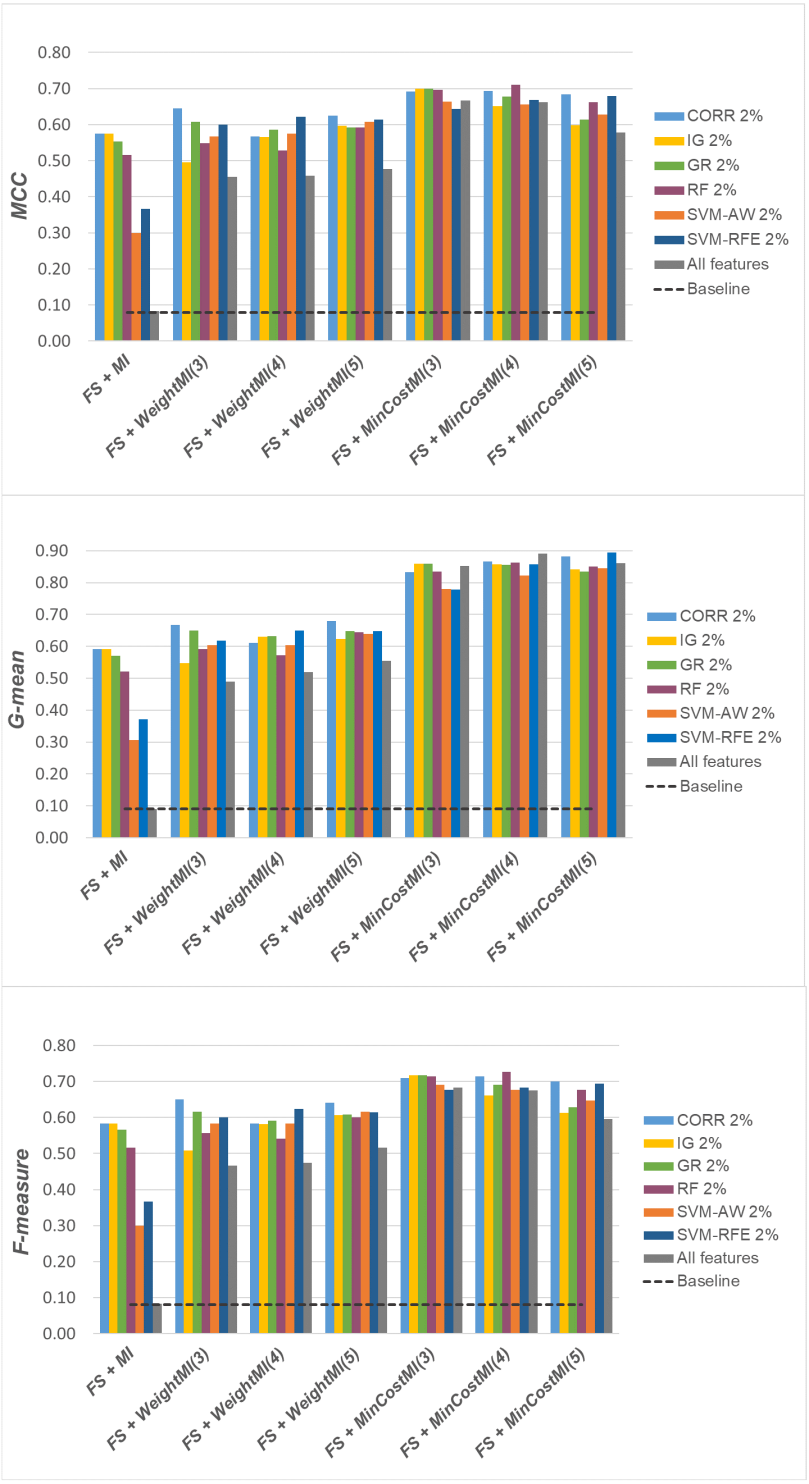
*Glioma* dataset: *MCC*, *G-mean* and *F-measure* performance achieved with different learning strategies, in conjunction with the six considered selection methods.

**Figure 11:**
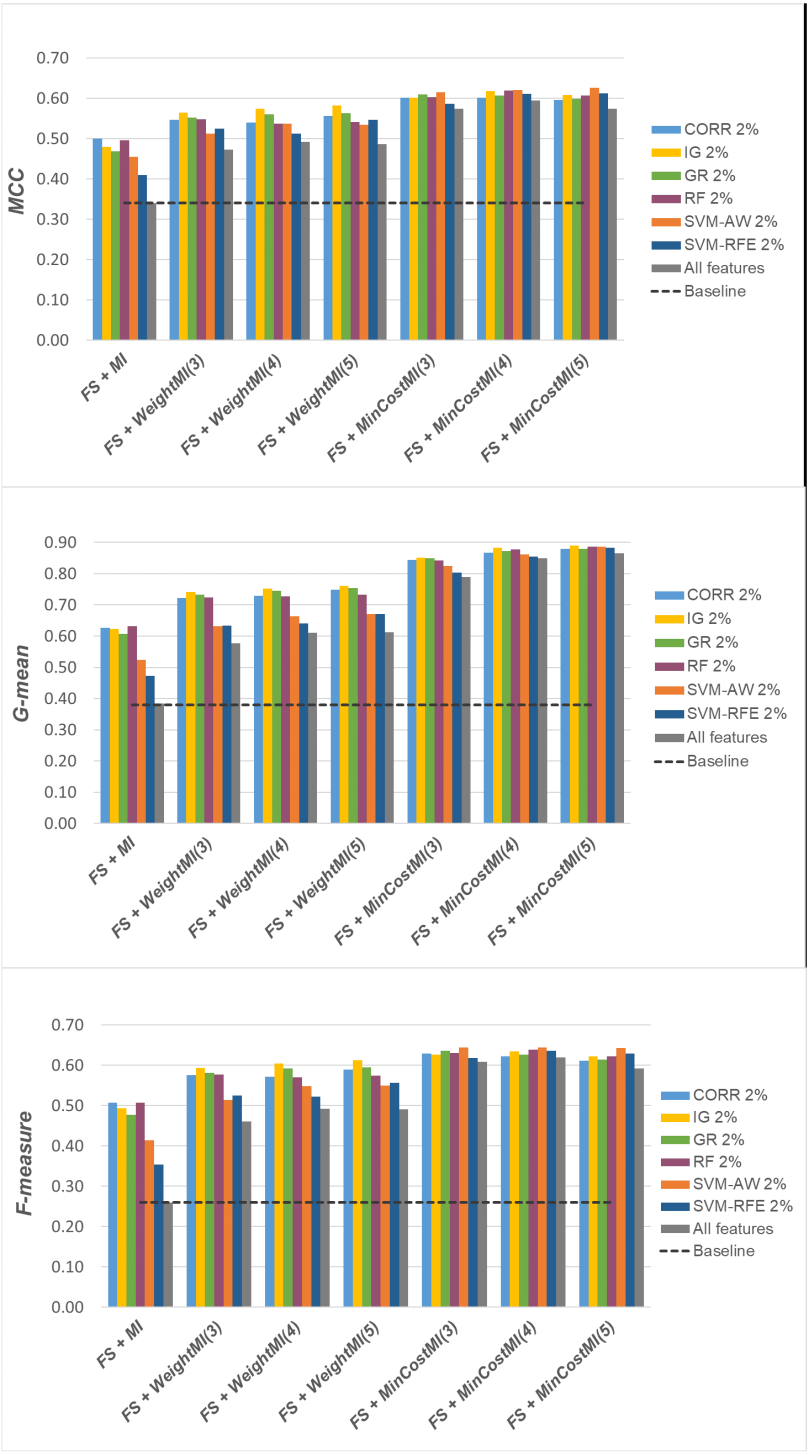
*Uterus* dataset: *MCC*, *G-mean* and *F-measure* performance achieved with different learning strategies, in conjunction with the six considered selection methods.

For a more complete picture, a second comparative view of our results is given in Figs. 9–11, where we focus on a given percentage of selected features (2%) and show the outcome of all the six selection methods considered in the study, in terms of *MCC*, *G-mean* and *F-measure*; for the sake of readability, the results of the *WeightFS+MI* strategy, less convenient than the others, have been here omitted. The performance of the other two strategies, *FS + WeightMI* and *FS+MinCostMI*, whose effectiveness has also been shown in Table 2, is here compared with that achieved *(i)* only using feature selection without cost-sensitive corrections, *(ii)* only using cost-sensitive learning without feature selection (data series ‘all features’ in the charts), and *(iii)* without using feature selection or costs (baseline). As we can see, although the obtained performance may depend on the specific selection method, as well as on the intrinsic data characteristics, the adoption of a hybrid learning strategy, that combines feature selection and cost-sensitive learning, is overall more convenient. When proper cost settings are used, indeed, it leads to the best results on the *DLBCL* datasets (Fig. 9). As regards the other two benchmarks (Figs. 10 and 11), the *MinCostMI* approach has proven to be capable of providing good results with and without feature selection. However, reducing the data dimensionality is of paramount importance in the considered domain, as well as in all domains where we need to acquire knowledge about the features that are most influential for prediction. Hence, we can still recommend the adoption of a hybrid learning strategy that allows to fully exploit the potential of cost-sensitive learning while using only a subset of the original features.

Albeit not exhaustive, the analysis here reported shows the importance of jointly addressing the issues of high-dimensionality and class imbalance and gives useful insight into how to introduce cost-sensitivity into the learning process, along with a proper dimensionality reduction step. Encompassing different selection heuristics, different levels of data reduction and different cost settings, this study complements related research works that have recently investigated the integration of feature selection and imbalance learning methods (Blagus & Lusa, 2013; Khoshgoftaar et al., 2014; Shanab & Khoshgoftaar, 2018; Zhang et al., 2019; Pes, 2020; Pes, 2021; Huang et al., 2021), showing that the hybrid learning strategies here explored may also be effective in challenging scenarios where the class imbalance problem comes in conjunction with very low instances-to-features ratios.

Differently from other works in this area, our approach is not tied to a specific selection or classification algorithm (Yin et al., 2013; Maldonado, Weber & Famili, 2014; Moayedikia et al., 2017; Feng et al., 2020), but relies on a general methodological framework that could be used as a meta-learning approach useful to integrate, and better exploit, a variety of methods already available. Interestingly, in the most imbalanced benchmarks, all the selection methods included in our study perform equally well when integrated into the *FS+MinCostMI* learning strategy, leading to similar results despite the significant differences observed when they are used alone. This seems to suggest that quite different selection heuristics can be successfully exploited within proper cost-sensitive methodological frameworks.

The effectiveness of the approach here discussed is also confirmed by a comparison with recent works in the literature (although the experiments are not always directly comparable due to the diversity of the evaluation protocols and metrics). In particular, our results compare well with those reported in recent papers that relied on data partitions involving 20% of test records, as in our study. For example, in terms of *F-measure* for the minority class, we achieved better results than (Yin et al., 2013) on the *DLBCL* dataset. In terms of *G-mean*, our results are superior to those reported in Moayedikia et al. (2017), where some selection methods designed for imbalanced data are compared. Our results are also comparable, in terms of *G-mean*, with the ones in Lin & Chen (2013), where different strategies for classifying high-dimensional and imbalanced data are explored, including ensemble correction strategies. Finally, our performance is only slightly inferior to the best results reported in Maldonado, Weber & Famili (2014) where, however, a different experimental protocol is used (*i.e.,* a leave-one-out cross-validation).

The encouraging results here obtained may pave the way for larger comparative studies involving more datasets from different domains. This could be very useful for researchers and practitioners in different application fields who might take advantage of methodological guidelines to deal with prediction tasks that involve both skewed data distributions and high-dimensional feature spaces.

## Conclusions & future research directions

In this work, we focused on challenging classification tasks where the imbalanced distribution of the data instances is coupled with a large number of features, which may severely impact on the generalization performance of commonly adopted classifiers. In such a context, we presented a comparative study aimed at exploring the extent to which different feature selection methods (both univariate and multivariate) may lead to a higher separability between majority and minority instances. Further, we explored different ways of integrating feature selection with cost-sensitive learning, by exploiting a methodological framework that is not tied to a specific selection algorithm or classifier.

The experimental analysis that we carried out on three public genomic benchmarks, encompassing different levels of dimensionality reduction and different cost settings, has provided some useful insight along the following directions:

• Feature selection, besides involving important advantages in terms of knowledge discovery and interpretability of the induced models, is also useful, in itself, in coping with the adverse effects of class imbalance, leading to a better separability among the different classes. In particular, in the presence of a moderate level of imbalance (as in the *DLBCL* dataset here considered), feature selection alone, without cost-sensitive corrections, leads to quite satisfactory results, not much inferior to those achieved with more sophisticated learning strategies.
• In the presence of a higher level of imbalance (as in the *Glioma* and *Uterus* datasets), additional benefits can be obtained, in terms of generalization performance, by integrating feature selection with cost-sensitive learning. Different ways of implementing such an integration have been here considered, by making the selection process cost-sensitive (*WeightFS + MI* learning strategy) or properly introducing costs at the model induction stage, after reducing the data dimensionality (*FS+WeightMI* and *FS+MinCostMI* strategies). Overall, the *FS+MinCostMI* approach has proven to be the most effective across the different settings explored in this study, leading to models that achieve good performance with a reduced number of features, irrespective of the specific selection algorithm employed. The strategy used for introducing costs into the learning process has therefore shown to be more influential than the specific selection heuristic chosen for implementation. Such an evaluation highlights the importance of devising proper learning strategies that integrate dimensionality reduction techniques and imbalance learning methods, to effectively deal with datasets that are both high-dimensional and class-imbalanced.

Starting from the analysis here presented, there are several aspects that we aim to explore in our future work. As a first point, it should be interesting to evaluate the impact of the learning strategies here investigated on different classifiers. For our experiments, indeed, we chose the *Random Forest* algorithm that has proven to be a suitable option across imbalanced classification tasks from different domains, as pointed out previously. But other choices could be also considered, so as to evaluate the extent to which different combinations of classifiers and selection methods may take advantage of the adoption of a cost-sensitive approach. Further, more benchmarks from different real-world domains will be analyzed to gain a deeper insight into the best strategies to integrate feature selection and cost-sensitive learning, based on the specific characteristics of the data at hand. In fact, making the classifier cost-sensitive has proven to be more effective, in the considered case study, than making the feature selection itself cost-sensitive, but further investigations could be conducted in this respect extending the evaluation to different feature selection approaches.

## Supplemental Information

10.7717/peerj-cs.832/supp-1 Supplemental Information 1 Datasets used in the experimental study The DLBCL dataset is in arff format (a link to the open repository where the original raw data is publicly available is included); the Glioma dataset in in arff format (a link to an open repository where the original raw data is publicly available is included); a link to an open repository where the Uterus dataset (over 100MB) is publicly available (in different formats, including arff) is included. For the analysis, we leveraged the WEKA machine learning workbench (freely available at https://www.cs.waikato.ac.nz/ml/weka/).

## Additional Information and Declarations

### Data Availability

The following information was supplied regarding data availability: The datasets are available in the Supplemental File.
